# Sex specific analysis of patients with and without reported statin intolerance referred to a specialized outpatient lipid clinic

**DOI:** 10.1186/s13293-024-00642-y

**Published:** 2024-09-02

**Authors:** Maximilian A. Muck, Marcus Fischer, Michael Hamerle, Christina Strack, Maxime Holzhaeuer, Dennis Pfeffer, Ute Hubauer, Lars S. Maier, Andrea Baessler

**Affiliations:** 1 Klinik und Poliklinik für Innere Medizin 2, University of Regensburg, Regensburg, Germany; 2 Caritas Krankenhaus St. Lukas, Kelheim, 93309 Germany

**Keywords:** Hypercholesterolaemia, LDL cholesterol goal attainment, Statin intolerance, Lipid lowering drugs, Sex and gender

## Abstract

**Background:**

Lowering LDL-cholesterol is a fundamental goal for both primary and secondary prevention of atherosclerotic cardiovascular diseases. Our study aims to analyse potential sex disparities regarding the tolerability and effectiveness of lipid-lowering therapy in patients with and without reported statin intolerance who are being treated at a lipid-outpatient clinic.

**Methods:**

From 2017 to 2022, *n* = 1062 patients (*n* = 612 men, *n* = 450 women) at high-risk were referred to our lipid-outpatient clinic because of difficulties in lipid control by primary healthcare providers. The main therapeutic objective was to optimize lipid-lowering therapy according to current treatment guidelines.

**Results:**

Patients presented with high LDL-C baseline levels (4.97 ± 1.81 mmol/l (192 ± 70 mg/dL) in men and 5.46 ± 2.04 mmol/l (211 ± 79 mg/dL) in women). Intolerance towards statins was reported more frequently by women (48.2%) than by men (38.9%, *p* = 0.004). LDL-C continuously decreased with individual treatment adjustments across follow-up visits. In total, treatment goals (LDL < 1.4 mmol/l (< 55 mg/dl) or < 1.8 mmol/l (< 70 mg/dl)) were accomplished in 75.8% of men and 55.5% of women after the last follow-up visit (*p* < 0.0001). In men, these data are almost identical in subjects with statin intolerance. In contrast, treatment goals were reached less frequently in women with statin intolerance compared to women tolerant to statin therapy.

**Conclusion:**

Even if treated in a specialized lipid clinic, women are less likely to reach their target LDL-C than men, particularly when statin intolerant. Nevertheless, many patients with statin intolerance can be successfully treated using oral combination and PCSK9 inhibitor therapy. However, ongoing follow-up care to monitor progress and to adjust treatment plans is necessary to reach this goal.

**Supplementary Information:**

The online version contains supplementary material available at https://doi.org/10.1186/s13293-024-00642-y.

## Abstract (plain language summary)

We investigated patients at high cardiovascular risk who were referred to our specialized lipid outpatient clinic because of elevated lipid levels and difficulties in lipid-lowering treatment in the primary care setting. The primary goal of such a clinic is to help patients to achieve optimal lipid levels through personalized treatment plans. We focused on prescription behavior and differences in treatment tolerability and effectiveness between men and women.

A large proportion of patients (more frequently women (48.2%) than men (38.9%)) reported intolerance towards statins and most patients’ LDL-cholesterol levels were far away from treatment goals. However, when treated at a specialized lipid clinic providing ongoing follow-up care to monitor progress and to adjust treatment plans if necessary, many of those patients were able to tolerate lipid lowering medication to achieve better lipid control and to maintain their lipid levels within target ranges.

However, women were less likely to reach LDL-cholesterol treatment targets compared to men, especially if they reported intolerance towards statins. Ongoing follow-up care to monitor progress and to adjust treatment plans is necessary to reach treatment goals.

**Supplementary Information:**

The online version contains supplementary material available at https://doi.org/10.1186/s13293-024-00642-y.

## Highlights

- If men or women are treated at a specialized lipid-outpatient clinic, a statin therapy can be implemented in many of those who were previously considered statin-intolerant.
- With the introduction of novel lipid-lowering drugs and combination strategies, it is possible to achieve treatment goals even with statin intolerance in a large proportion of patients.
- Despite receiving a treatment at a specialized lipid center, women were less likely to reach their target LDL-C.

**Supplementary Information:**

The online version contains supplementary material available at https://doi.org/10.1186/s13293-024-00642-y.

## Introduction

Elevated LDL cholesterol (LDL-C) levels are a major risk factor for atherosclerotic cardiovascular diseases (ASCVD) [1]. Thus, lowering LDL-C is the fundamental therapeutic objective for primary and secondary prevention of cardiovascular (CV) diseases [2–7]. In fact, reduction of LDL-C with statins reduces the risk of major ASCVD events, including all-cause and CV mortality, largely irrespective of sex, age, and baseline LDL-C levels, even in people at low risk [7] and in the elderly [8]. Some studies suggest that women may benefit slightly less from high-intensity statin therapy compared to men, though the overall impact remains substantial. However, women are often underrepresented in clinical trials, leading to less robust data specifically addressing sex differences in response to statin therapy. ​The benefit of LDL-C lowering has also been clearly demonstrated using other emerging non-statin pharmacologic treatment options, [9–11] and existing knowledge suggests that these lipid lowering agents are equally effective in men and women [12, 13]. 

The 2019 ESC/EAS guidelines for the treatment of dyslipidaemias advise target-values according to cardiovascular risk categories. Patients at very high risk, i.e. with established ASCVD, familial hypercholesterolaemia (FH) with additional CV risk factors, diabetic end organ damage, or advanced chronic kidney disease, aim to reduce their LDL-C levels below the absolute target < 1.4 mmol/l (< 55 mg/dl), in addition to achieve a relative ≥ 50% reduction in LDL-C from baseline.

These treatment goals are identical for men and women [3]. 

In spite of established clinical practice guidelines on CV risk prevention and dyslipidaemias, recent surveys assessing the use of lipid-lowering therapy (LLT) and attainment of LDL-C targets found only suboptimal overall goal achievement in European and US patients, particularly in those at high CV risk and in women [14, 15]. Exemplarily, a recent analysis of over 600,000 patients with ASCVD indicated that approximately half were not taking statin therapy, and only 22.5% were on high-intensity statins [16]. This underutilization of appropriate lipid-lowering therapies contributes to suboptimal LDL-C control​. The GOULD registry, which tracked patients with ASCVD over two years, showed that LDL-C target attainment remains poor, with a significant proportion of patients not reaching the recommended levels, particularly women [17]. This registry highlighted the need for more aggressive and comprehensive lipid management strategies, including the use of combination therapies.

In fact, according to several reports it is apparent that women have worse control of cardiovascular risk factors, in particular hypercholesterolemia, than their male counterparts [18–22]. However, whether this sex disparity is due to biological, behavioral, or both factors, is not clear.

With the introduction of modern and effective LLT additional to statins, such as PCSK9 inhibitors, bempedoic acid, and ezetimibe, and moreover, the possibility of utilizing nearly any combination therapy, a more effective pharmacological armamentarium exists to better achieve treatment targets, even in patients with statin intolerance. Indeed, it has been repeatedly demonstrated that combination therapy is effective and superior to monotherapy for many patients [23–26]. 

Whether lipid control is improved in challenging-to-treat patients at high CV risk in an outpatient lipid clinic implementing personalized modern treatment algorithms in statin tolerant and intolerant male and female patients is the central question of this study. Thus, we conducted a focused analysis using data from our lipid-outpatient clinic registry to examine sex -related differences in presentation, treatment, and response, with the aim of evaluating sex disparities in the attainment of LDL-C goals.

## Materials and methods

### Study population

Patients with lipid disorders or patients at high cardiovascular (CV) risk who were referred to a specialized lipid outpatient clinic at the University Hospital of Regensburg, Germany, were entered into an on-site lipid care quality control database. All patients with an initial visit between 2017 and 2022 were included (*n* = 1062 patients, *n* = 612 men, *n* = 450 women) and retrospectively analysed. All patients were identified by their sex assigned at birth, as either male or female. Reasons for referral were the need for improvement of LLT, intolerance towards lipid-lowering drugs, inability to reach the target values for LDL-C or triglycerides, initiation of PCSK-9 inhibitor therapy, genetic testing for familial hypercholesterolaemia (FH) or because elevated LDL-C was spotted in the family. These patients were considered challenging to treat.

All patients were encouraged to participate in their routine clinic follow-up visit.

The following data were collected from medical records and questionnaires: demographic characteristics, past medical history, height, weight, and blood pressure; recent lipid values recorded within 12 months prior to (and including) the enrolment visit; LLT at the enrolment visit and in the preceding 12 months; detailed history of intolerance to any statin at any dose; reasons for LLT prescription in patients without previous atherosclerotic events, concomitant medications, and the FH Score in order to classify FH. The FH Score is intended for use by healthcare professionals to assist in the diagnosis of adult patients at risk of heterozygous FH. It is based on the guidelines of the Consensus Statement of the European Atherosclerosis Society. By answering the questions known as the Dutch Lipid Clinic Network (DLCN) criteria, the score can be used to calculate the probability of an FH diagnosis as unlikely (FH Score 0–2), possible (FH Score 3–5), probable (FH Score 6–8) or definite FH (FH Score > 8). Here, an FH Score > 8 and/or a positive genetic test result was used to *define FH*.

*Statin intolerance* was defined as adverse effects (muscle pain, elevation of creatine kinase or liver enzymes and gastrointestinal adverse events such as constipation, abdominal pain, or diarrhoea) associated with the intake of at least two different statins which resolve or improve with dose reduction or discontinuation and with inability to tolerate the dose necessary to achieve the patient-specific therapeutic objective.

Hypertriglyceridemia was defined as a triglyceride value above the threshold of 1.7 mmol/l (150 mg/dl), hypercholesterolaemia as an LDL-C value that did not reach the individual target area according to the 2019 ESC/EAS guidelines for dyslipidaemia management. Patients with combined dyslipidaemia met both criteria.

### Data collection

A minimum of one follow-up visit, a maximum of six visits were recorded. The time span between presentations for one patient typically ranged from three months to one year. Collected data at each visit included anthropometric information, cardiovascular risk factors, cardiovascular diseases that occurred before the first visit or between presentations, FH-Score, LLT before and after each presentation, drug intolerances having occurred before the first visit or between presentations, LDL-C level (which was measured photometrically) at each visit. Comorbidity data were obtained using survey interviews and consecutively verified by medical record entries. Coronary Artery Disease (CAD) was defined as history of myocardial infarction or coronary revascularization procedures (PCI and/or CABG) and/or presence of at least one angiographically documented coronary stenosis ≥ 50%. Cerebrovascular Disease was a history of stroke, TIA, carotid endarterectomy/angioplasty or the presence of a carotid stenosis of ≥ 50% identified through imaging studies such as carotid ultrasound or angiography. Peripheral Artery Disease (PAD) was defined as history of peripheral revascularization procedures such as angioplasty or surgery for iliac and/or lower-extremity arteries or a diagnosis of intermittent claudication.

According to the ESC/EAS guidelines, the risk categories were designated and the main objective of the therapy was to reach the associated target LDL-C recommended for each patient. The study was approved by the institutional ethical review board at the University of Regensburg, Germany (EK number 23-3265-104).

### Statistical analysis

Statistical analysis was performed with JMP 17 (SAS Institute, Cary, NC, USA) and Stata Statistical Software, Release 14, College Station, TX: StataCorp LLC). The values are presented as counts and percentages or means ± standard deviations. Fisher’s exact test was used to identify differences between men and women in baseline categorical variables. The Shapiro-Wilk test for normality was used to examine continuous variables for normality. For normally distributed continuous variables, the Student’s t-test was used to assess mean differences between both sex groups, and the nonparametric Wilcoxon-Mann-Whitney U test was applied when the data have not met the assumption of normality. We used linear mixed-effects models with fixed and random effects to analyse sex effect on repeated measurements of LDL-C levels across follow-up visits to construct mixed models with unequal timing between repeated measurements across experimental units or individual patients. Here, sex category was set as fixed effect and individuals nested in sex categories were set as random effects, allowing for multiple sources of variability within the data to be captured in the mixed-effects model. The method of Generalized Estimating Equations (GEE) was used to analyse discrete dependent variables (i.e. achievement of LDL-C targets, yes or no) that are measured repeatedly across follow-up visits. A *p-*value of < 0.05 was reported as statistically significant.

## Results

Baseline characteristics were documented for *n* = 1062 patients of whom *n* = 612 were male and *n* = 450 were female (see Table 1). Women showed higher LDL-C, HDL-C, and total cholesterol levels, whereas men had a higher BMI, had been diagnosed with hypertension more often and displayed higher triglyceride values compared to women. The diagnosis of isolated hypercholesterolaemia and heterozygous FH was established more often for women, hypertriglyceridaemia and combined dyslipidaemia more often for men. There was no significant difference regarding age, smoking, as well as the frequency of diabetes, and Lp(a) elevation in the patient cohort.

**Table 1:** Baseline characteristics of Study Population

	Men (*n* = 612)	Women (*n* = 450)	*p*-value
Age, yrs.	54 ± 14	56 ± 14	0.014
BMI, kg/m2	28.6 ± 4.6	26.5 ± 5.4	< 0.0001
Hypertension, n (%)	378 (61.8)	205 (45.7)	< 0.0001
Smoking, n (%)	181 (32.2)	107 (24.8)	0.011
Diabetes, n (%)	113 (18.5)	57 (12.7)	0.011
Total-C, mg/dL	219 ± 72	257 ± 75	< 0.0001
LDL-C, mg/dL	190 ± 71	208 ± 78	< 0.0001
HDL-C, mg/dL	48 ± 15	63 ± 18	< 0.0001
Non-HDL-C, mg/dL	167 ± 74	193 ± 76	< 0.0001
Triglycerides, mg/dL	324 ± 442	187 ± 166	< 0.0001
Hypercholesterolaemia, n (%)	411 (67.4)	367 (81.9)	< 0.0001
Combined hyperlipidemia, n (%)	196 (32.1)	78 (17.4)	< 0.0001
HetFH, n (%)	37 (6.1)	44 (9.9)	0.023
Lp(a), mg/dL	53 ± 55	63 ± 62	ns
Lp(a), nmol/L	132 ± 131	138 ± 129	ns
Lp(a) increased*, n (%)	163 (26.6)	152 (33.8)	ns

Total study population (*n* = 1062, of these *n* = 996 patients at very high- or high-risk were included for further analyses); BMI-body mass index; C-cholesterol; HetFH-heterozygote familial hypercholesterolemia; Lp(a)-lipoprotein(a);* ≥30 mg/dL or ≥ 75 nmol/L

The majority of patients were categorized into very high or high cardiovascular risk groups according to 2019 ESC/EAS guidelines on dyslipidaemias [3]. Specifically, 82.2% of male patients and 71.3% of female patients were placed within the very-high-risk group, 20.4% of women and 13.1% of men within the high-risk group. Only 4.7% of men and 8.2% of women were classified as moderate or low risk patients (and were excluded from further analysis).

Overall, in the constituted final study population of 996 men and women at very high or high CV risk according to 2019 ESC/EAS guidelines CV disease was present more often in men than in women. CAD (coronary artery disease) was the most common cardiovascular disease manifestation in both men (53.9%) and women (29.8%) followed by cerebrovascular disease (17.2% of men and 14.5% of women) as well as diabetes mellitus (18.9% of men and 12.4% of women). 8.4% of male patients and 6,1% of female patients had a known diagnosis of symptomatic peripheral artery disease.

**Fig. 1:**
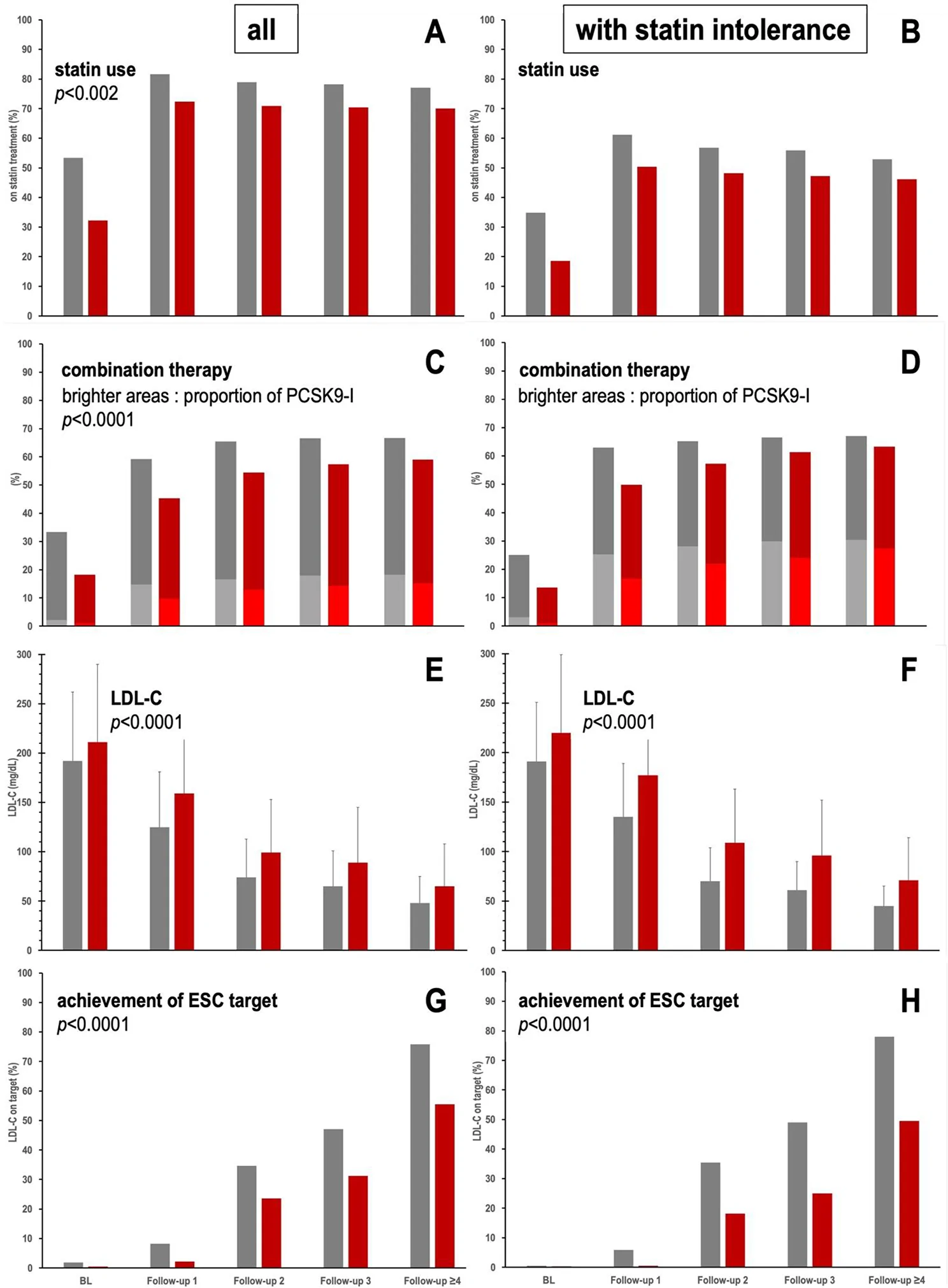
Title: Sex disparities in treatment patterns and goal attainments: analysis across clinical follow-up visits. Legend: Overview of treatment patterns, LDL-cholesterol levels, and goal attainments according to European Society of Cardiology/European Atherosclerosis Society (ESC/EAS) 2019 risk categories and treatment goals for LDL-cholesterol in very-high and high-risk patients, across subsequent clinical follow-up visits. Data shown are for male (grey bars) and female (red bars) patients considered in the overall group of patients (*n* = 996, left figures, panels **A**, **C**, **E**, **G**) and in patients with reported statin intolerance (*n* = 426, right figures, panels **B**, **D**, **F**, **H**). *P*-values indicate overall statistical significance in differences between women and men and were derived by linear mixed-effect models for the continuous parameter LDL-C and Generalized Estimating Equations for dichotomous parameters. (**A**, **B**) percentage of patients using statins, (**C**, **D**) percentage of patients using combinations of lipid-lowering drugs, (**E**, **F**) mean LDL-cholesterol levels, (**G**, **H**) percentage of patients who reached ESC/EAS treatment goals. Treatment goals for very-high risk (*n* = 825) and high risk patients (*n* = 171) were different according to ESC/EAS guideline recommendations. The number of visits for each patient and the time span between visits may differ. Statin intolerance was defined as adverse effects associated with the intake of at least two different statins which did resolve or improve with dose reduction or discontinuation. Combination lipid lowering therapy includes a statin and at least one other lipid lowering agent, that isn´t a statin such as ezetimibe, bempedoic acid, evolocumab, alirocumab, or inclisiran

At the baseline visit, only 53.4% of men and 32.2% of women with very or high CV risk were treated with statins (Fig. 1, panel A, *p* < 0.0001). At this point, intolerance to statins was reported more frequently by women (48.2%) than by men (38.9%, *p* = 0.004). In contrast, women complaining of side effects of statin therapy had less frequently elevated creatine kinase levels than men (9.1% vs. 20.7%, *p* = 0.008). Accordingly, the use of statins in patients with some degree of statin intolerance was 34.8% in men and 18.6% in women (Fig. 1, panel B, *p* < 0.0002). Statin intolerance was more common in very high risk than in high risk patients (50.5% vs. 28.2%, *p* < 0.0001) and the last documented dosages of statins before referral to our outpatient clinic were higher in very high risk patients than in high risk patients (i.e. atorvastatin: 48.7 ± 24.8 vs. 28.0 ± 17.2 mg/day, rosuvastatin: 20.9 ± 13.1 vs. 12.5 ± 8.1 mg/day, simvastatin: 38.1 ± 20.6 vs. 24.2 ± 17.4 mg/day, each *p* < 0.001). After the first visit in our outpatient lipid clinic, statin therapy was prescribed in 81.6% of men and 72.4% of women (Fig. 1, panel A) and in 61.2% and 52.2% of men and women with reported statin intolerance (Fig. 1, panel B). Despite the high prevalence of statin intolerance reported initially, the frequency of statin treatment remained almost unchanged during the subsequent follow-up visits. In parallel, the frequency of combination LLT increased during this time (Fig. 1, panel C and D). However, the proportion of statin use and the use of combination LLT was consistently lower in women than in men. Detailed information regarding lipid-lowering therapies, particularly statin treatment (including dosages), are available in supplementary Tables 1–8.

Aiming to reach ESC treatment targets, PCSK9 inhibitors were used increasingly across follow-up visits, particularly in subjects with statin intolerance (Fig. 1, panel C and D). In fact, in the group of very high-risk patients with LDL-C target < 1.4 mmol/l (< 55 mg/dL, data not shown) PCSK9 inhibitors were prescribed in almost half of patients with statin intolerance (49.3% of men and 44.5% of women) after at least four follow-up visits.

At baseline, mean LDL-C levels were elevated in patients referred to our specialized outpatient lipid clinic: 4.97 ± 1.81 mmol/l (192 ± 70 mg/dL) in men and 5.46 ± 2.04 mmol/l (211 ± 79 mg/dL) in women at high or very high risk. LDL-C continuously decreased across follow-up visits. Mean LDL-C reduction to < 1.4 mmol/l (< 55 mg/dl) could be achieved on average after 4 subsequent visits in men (mean LDL-C reduction to 1.24 ± 0.7 mmol/l (48 ± 27 mg/dL)). This LDL-C goal could not be accomplished on average in women (mean LDL-C reduction to 1.68 ± 1.11 mmol/l (65 ± 43 mg/dL), Fig. 1, panel E). In subjects with statin intolerance, LDL-C levels declined more slowly or somewhat delayed, but at the end they were not different from total population LDL-C levels, probably due to the higher proportion of PCSK9-inhibitor treatment in subjects with statin intolerance (Fig. 1, panel F and D). By the time of the last documented visit, men had reduced their LDL-C by 74 ± 15% and women by 69 ± 17% (*p* = 0.0013). The sex disparity in LDL-C reduction (in percentage), with women experiencing a less pronounced decrease compared to men, remained consistent regardless of the frequency of visits or the varying time intervals between those visits (supplemental figure).

The achievement of ESC LDL-C target values in male and female subjects with and without reported statin intolerance is shown in Fig. 1, panel G and H. In total, treatment goals (LDL < 1.4 mmol/l or < 1.8 mmol/l, respectively) were accomplished in 75.8% of men and 55.5% of women after the last documented follow-up visit (*p* < 0.0001). The additional treatment goal of lowering LDL-C by at least 50% was also reached more often in men than in women (93.6% of men and 85.8% of women, *p* < 0.05). In men, these data are similar in subjects who complained of inability to tolerate statins at the prescribed dosage (Fig. 1, panel F). In contrast, treatment goals were reached less frequently in women with statin intolerance compared to women tolerant to statin therapy. Of note, more men than women reached their ESC treatment targets (Fig. 1, panel G and H). These differences might be due to a lower proportion of statin use in women who do not reach their treatment goals (supplementary Tables 7–8).

## Discussion

Low-density‐lipoprotein cholesterol (LDL‐C) is accepted as a causal risk factor for the development and progression of atherosclerotic cardiovascular disease (ASCVD) and its resulting CV disease events, such as myocardial infarctions [1]. Thus, LDL-C lowering is an integral part of all current guidelines on the treatment of dyslipidaemias and on ASCVD prevention and treatment [2, 3, 27]. 

Statins are the first-line therapy for managing elevated cholesterol levels in individuals with elevated CV risk, based on their efficacy in reducing major CV disease events and mortality [2, 3, 28]. They are one of the most prescribed drugs and are generally well tolerated. However, despite the convincing evidence from numerous randomized controlled trials, non-adherence to treatment and discontinuation of statin therapy frequently occurs in clinical practice and is associated with worse outcomes due to an increase in CV disease events [29–33]. 

Therefore, LDL-C treatment goals often remain unattained. In fact, according to a recent systematic review including > 300,000 patients from 81 observational studies, [34] as well as according to the EUROASPIRE IV, [22] V, [35] and the DA-VINCI surveys, [14] achievement of LDL-C targets was poor among patients with established ASCVD. In view of the current available pharmacotherapeutic options, this poor treatment target achievement is remarkable since, according to a simulation study, 9 out of 10 post myocardial infarction patients were able to achieve the recommended LDL-cholesterol goals using the available lipid-lowering drugs [36]. 

Patients frequently discontinue statin therapy without medical advice because of perceived side effects, particularly myalgia, among others. However, undertreatment has been considered a multifactorial phenomenon, in which conditions related not only to patients, but also physicians, public healthcare systems, and media, mutually interact.

Perhaps some of these factors could be overcome by the implementation of extra patient care within specialized outpatient lipid clinics in order to help patients achieve and maintain optimal lipid levels through personalized plans, follow-up care with treatment adjustments as needed, and lifestyle modifications to reduce CV risk. For example, if patients report intolerance to statin treatment it may be advisable to change the dose, switch to a different statin, or try an alternate-day regimen, use of combination therapy, or if intolerance persists even at the lowest dose, non-statin drugs should be considered. This is in accordance with results of the PALM registry demonstrating that patients who have refused statin treatment due to concerns about side effects were willing to reconsider statin therapy if offered [37]. However, such an approach requires time, repeated follow-up visits and an ongoing and stable process of patient-physician relationship.

The present analysis aimed to explore whether specialized care in an outpatient lipid clinic implementing modern treatment algorithms improves lipid control in difficult-to-treat patients at high CV risk and assessed factors related to lack of attainment of treatment goals. We found that in these patients referred to our institution, intolerance towards statins was common and was reported more frequently by women (48.2%) than by men (38.9%). However, our findings illustrate that a statin therapy can be implemented at least at low-dosage in a large proportion of those who were previously considered statin-intolerant, and this was more common in men than in women. This may lead to the conclusion that the diagnosis of statin-intolerance is often implemented too early without starting further therapy attempts using an alternative statin or with a statin in a lower dosage. In accordance with our findings, a recent meta-analysis, the largest study to investigate this question comprising > 4 million patients from 176 studies, showed that the true prevalence of statin intolerance is low (between 6 and 10% worldwide) and clearly demonstrated the overestimation of statin-intolerance [38]. 

Thus, these and our findings imply that patients` symptoms related to statins should be evaluated carefully, firstly to see whether symptoms are indeed caused by the drug, and secondly, to evaluate whether symptoms are rather due to patients’ perceptions that statins are harmful, the so-called nocebo effect.

Another common feature of our results with those of the meta-analysis as well as other reports in the literature is that females are more likely to be statin intolerant. Notably, female sex was the most important predictor of statin intolerance [38, 39]. Due to the fact that female sex turned out to be the most relevant predictor of statin intolerance, differences in perception and drug metabolism and tolerability must be assumed. Given the frequently higher plasma concentration of statins, possibly due to the smaller body size, the lower glomerular filtration rate, and the higher proportion of body fat, there is a biological explanation for why women experience more side effects than men. Thus, women are nearly twice as likely to discontinue statin therapy due to adverse effects [40–42]. Despite similar treatment guidelines, several studies have shown that women have worse control of LDL-C levels than their male counterparts [18–22]. Besides the above listed biological reason of a more prevalent statin intolerance, this could be attributed to additional factors, particularly a decreased medication adherence in women. Indeed, numerous studies - including a comprehensive meta-analysis of 53 studies - have demonstrated that adherence is markedly lower among women compared to men [38]. Moreover, underestimation of cardiovascular disease risk in women, and decreased prescription and utilization of lipid-lowering therapies, are further factors resulting in lower attainment of lipid targets in women. It is therefore imperative for clinicians to address statin intolerance and medication adherence to eliminate sex-specific differences in attainment of lipid goals.

In case of assured true statin intolerance other treatment options are available, such as bempedoic acid, ezetimibe, and PCSK9 inhibitors, and effective to lower LDL-C to treatment targets. However, little is known about the ability to achieve LDL-C targets in CV risk patients who cannot tolerate any statins at any dose or at a dose required to reduce LDL-C sufficiently from their baseline levels. Here we show that treatment goals could be accomplished in a significant proportion of patients with reported statin intolerance, at least in men.

### Strength and limitations

Our data comprehensively capture the unique clinical course of each individual patient, emphasizing the importance of personalized management. It should be noted that treatment effects could be influenced by the varying number of follow-up visits for each patient. Furthermore, our study focused on a challenging and complex patient population with difficult-to-treat conditions, encompassing a substantial number of high-risk individuals with elevated LDL-C or triglyceride levels, familial hypercholesterolaemia, hyperlipoproteinemia (a) and statin intolerance. Therefore, the results of our analyses are not fully transferable to all patients with hypercholesterolaemia. As most of the patients were classified as being at very high risk (target LDL-C < 55 mg/dL) and only a small percentage (17%) as being at high risk (target LDL-C < 70 mg/dL) we did not analyse the achievement of LDL-C treatment goals separately in both subgroups by sex. However, the achievement of LDL-C goals was comparable in very high- and high-risk patients (67% vs. 70%, n.s.).

Moreover, since it is known that cholesterol metabolism is influenced by sex hormones, our results may be affected by different life phases, such as pre- and post-menopause in women. However, due to the inclusion of female patients with familial hypercholesterolemia, which biases the usual LDL-C rise with increasing age, we have not conducted any additional subgroup analyses dividing women into these categories.

It should be emphasized that the treatment strategy remained consistent across all patients as the data were collected from the same specialized outpatient clinic. This ensured adherence to ESC/EAS guidelines and maintained the uniformity of treatment escalation for each patient, allowing for meaningful comparability in the study.

### Perspectives and significance

Statin intolerance continues to pose a significant obstacle in achieving target LDL-C levels, particularly among women. In difficult-to-treat patients ongoing follow-up care is necessary to reach LDL-C treatment goals.

## Conclusions

Even if treated in a specialized lipid clinic, women are less likely to reach their target LDL-C than men, particularly when statin intolerant. Nevertheless, a large proportion of patients with statin intolerance can be successfully treated using oral combination and PCSK9-inhibitor therapy. However, strict monitoring with several follow-up visits and treatment modifications is necessary to reach this goal.

## Electronic supplementary material

Below is the link to the electronic supplementary material.

Supplementary Material 1

## Data Availability

The data sets generated and/or analysed during the current study are not publicly available due to ongoing studies but are available from the corresponding author on reasonable request. Previous presentations of the work presented in the article: not performed.
