# Supplementary material for: Sex specific analysis of patients with and without reported statin intolerance referred to a specialized outpatient lipid clinic
Source: Biol Sex Differ. 2024 Sep 2;15:67. doi: 10.1186/s13293-024-00642-y (PMC11367976; doi:10.1186/s13293-024-00642-y)
Supplement: Supplementary file 1 — Supplementary Material 1 [file 13293_2024_642_MOESM1_ESM.docx]

Prof. Dr. Andrea Baessler, MD

Clinic for Internal Medicine 2

University of Regensburg, Germany

Jill Becker, PhD

Chief Editor

Biology of Sex Differences

July 7^th^, 2024

Dear Editors,

I hope this letter finds you well. We are pleased to resubmit our revised manuscript entitled **"Sex specific analysis of patients with and without reported statin intolerance referred to a specialized outpatient lipid clinic"** for consideration of publication in *Biology of Sex Differences*. We greatly appreciate the detailed feedback provided by the reviewers and the editorial board.

In response to the reviewers' comments, we have made substantial revisions to the manuscript. We have included a point-by-point response within the 'Response to Reviewers' box in the submission system, addressing each comment and suggestion.

We have ensured that our revised manuscript conforms to the journal style, as detailed in the Submission Guidelines on the journal homepage.

Thank you for your guidance and the opportunity to improve our manuscript. We are convinced that the paper has been substantially improved by the review process. We look forward to your feedback and hope that our revised submission meets the high standards of *Biology of Sex Differences*.

Sincerely,

Prof. Dr. Andrea Baessler, MD

Clinic for Internal Medicine 2

University of Regensburg, Germany
